# Locally Invasive Pancreatic Adenocarcinoma Presenting in the Splenic Flexure: A Rare Case Report

**DOI:** 10.7759/cureus.8085

**Published:** 2020-05-13

**Authors:** Lakmal S Ekanayake, David Jenkins, Stephen Swade, Harsh P Patel, David Bruce

**Affiliations:** 1 Medicine, Ohio University Heritage College of Osteopathic Medicine, Athens, USA; 2 General Surgery, Grandview Medical Center, Dayton, USA; 3 Medicine, University of Cincinnati College of Allied Health Sciences, Cincinnati, USA

**Keywords:** pancreatic adenocarcinoma

## Abstract

Pancreatic adenocarcinoma is a malignant cancer seen predominantly in males presenting with high-risk factors including chronic pancreatitis, familial history of cancer, and tobacco and alcohol abuse. The etiology of pancreatic adenocarcinoma is deceptive, and research continues to investigate its molecular patterns. Herein, we present a case of a 74-year-old Caucasian male who presented to the emergency department (ED) with tarry stools and hematochezia in the rectum for two weeks. Colonoscopy was terminated prematurely due to a mass at 70 cm within the splenic flexure of the colon. Exploratory laparotomy revealed a palpable mass at the splenic flexure invading the spleen. Splenectomy, distal pancreatoduodenectomy, and left hemicolectomy were performed. Pathological imaging revealed locally invasive pancreatic adenocarcinoma presenting in the splenic flexure, pancreatic parenchyma, peripancreatic soft tissue, and colonic wall. The patient is currently undergoing chemotherapy and radiation treatment. Below, we discuss risk factors, pathology, screening methods, and current treatment guidelines regarding pancreatic cancer. When pancreatic adenocarcinoma becomes metastatic, it most commonly involves the liver and lungs, but the review of current literature shows that limited cases of local invasion to the splenic flexure have been reported.

## Introduction

Pancreatic cancer is the third leading cause of cancer mortality in the United States, following lung and colon cancer respectively [1]. The etiology of pancreatic adenocarcinoma is predominantly focused on genetic inheritance and mutations. Environmental factors in disease pathogenesis are well documented and include obesity, tobacco, alcohol, chronic pancreatitis, and diabetes mellitus. Some of the non-modifiable risk factors include metabolic syndrome, diabetes mellitus and hereditary forms of chronic pancreatitis [1]. Current diagnostic and screening protocols remain poor and, ultimately, these cancers are found late with poor prognosis. Metastasis of pancreatic cancer can occur in severe cases, commonly to the liver, lung, and sigmoid colon [1]. Once diagnosed, chemotherapy, radiation, and surgery are the only treatment options. These treatment strategies remain poor and have low success rates.

The current treatment strategy includes a chemotherapy regimen and surgery for advanced disease [2]. New therapies of immunomodulators that target the microsatellite instability pathway have been brought to the market but cause a significant amount of financial strain on patients of up to $100,000 per year [2].

The current five-year survival rate for pancreatic adenocarcinoma is 2%-9%, with a geographic predominance in developed countries [3,4]. With this global trend, the rise of pancreatic cancer is slated to increase to the second most common cause of cancer-associated deaths in the United States [3].

By investigating key molecular patterns, current research has explored the genome and epi-genome profile of pancreatic cancer. Screening protocols have drastically increased our understanding of the development of pancreatic cancer. These histologic precursors include pancreatic intraepithelial neoplasia (PanINs), intraductal papillary mucinous neoplasms, and mucinous cystic neoplasm [5]. By investigating these molecular patterns, the hope is to detect cases earlier, provide the most appropriate treatment strategies, and improve outcomes.

## Case presentation

A 74-year-old Caucasian male presented to the emergency department (ED) with chief complaints of tarry stools and hematochezia in the rectum. The patient stated that he noticed a change in his bowel movements and significant lethargy and fatigue over the past two weeks. Significant medical history included persistent atrial fibrillation, ischemic cardiomyopathy, essential hypertension and type two diabetes mellitus with stage 2 chronic kidney disease. Surgical history included coronary stent placement and cardioverter-defibrillator. Current medications include rivaroxaban, atorvastatin, hydrochlorothiazide, lisinopril, metoprolol, metformin, and pioglitazone. Significant social history includes alcohol and tobacco abuse. 

Initial workup within the ED included complete blood count (CBC) with differential, computed tomography (CT) of the abdomen/pelvis without contrast, chest X-ray, and ultrasound of the abdomen (Table 1). All imaging was non-contributory, and the patient was admitted for gastroenterology consultation due to gastrointestinal bleeding. 

**Table 1:** Abnormal lab values presenting in the emergency department RBC: red blood cells; HGB: haemoglobin.

Category	Value	Reference Range
RBC	2.15	4.30-5.86 M/uL
HGB	7.1	13.1-17.6 g/Dl
Blood Urea Nitrogen	33	7-18 mg/dL
Creatinine	2	0.6-1.3 mg/dL

Upon consultation, the diagnostic plan consisted of esophagogastroduodenoscopy (EGD) and colonoscopy to assess the source of the bleed. EGD showcased mild gastritis without evidence for upper gastrointestinal bleeding. Colonoscopy showcased two ulcers at the distal transverse/splenic flexure and an obstructive mass in the descending and sigmoid colon at approximately 70 cm. This mass prevented the further advancement of the scope. A biopsy was obtained, and pathology showcased fragments of benign colonic mucosa with ulceration, moderately differentiated adenocarcinoma within the sigmoid colon, and hyperplastic polyps of the sigmoid and rectum. Lynch syndrome proteins (MSH2, MSH6, MH1, and PMS2) were tested and were found to be normally expressed. 

Due to the obstructive mass, general surgery was consulted for exploratory laparotomy. During intraoperative exam, a mass was palpable at the splenic flexure which appeared to invade the nearby spleen. Respectively, a left hemicolectomy, splenectomy, and a partial distal pancreatectomy were performed. A significantly enlarged mesenteric lymph node near the transverse colon was found and resected. 

Surgical biopsy and cancer markers demonstrated pancreatic ductal adenocarcinoma extending into the wall of the splenic flexure (Table 2). It was noted that the carcinoma involved the pancreatic parenchyma, peripancreatic soft tissue, colonic wall, and thirteen lymph nodes. Cancer staging was T2N2M0.

**Table 2:** Antibody tests CA: carbohydrate antigen; DAT Anti-IgG: direct antiglobulin test with anti-immunoglobulin G.

Category	Value	Reference Range
CA 19-9	1012.90	0-35
Lactate Dehydrogenase	470	110-270
DAT Anti-IgG	Negative	Negative

## Discussion

Pancreatic adenocarcinoma has the highest mortality rate of all cancers, with a five-year prognosis of 2%-9% because it characteristically presents late in the course of the disease [5]. Lack of screening method contributes to the increased mortality rate due to late diagnoses. In a study of 225 high-risk patients, endoscopic ultrasound examination (EUS) found the highest percentage of pancreatic abnormalities with a sensitivity of 42.6%, followed by magnetic resonance imaging (MRI) at 33.3% and CT at 11.0% [6]. CT and MRI lack the capability of obtaining a tissue biopsy, making EUS the modality of choice for diagnosis [7]. Screening in asymptomatic patients remains unadvised due to potential harms outweighing potential benefits [7]. ­­

Pancreatic cancer is often deadly and the only reliable treatment to cure pancreatic cancer is surgical resection with adjunct chemotherapy to improve long-term survival. The difficulty surrounding complete surgical resection is attributed to the cancer having metastasized at the time of diagnosis [8]. Pancreaticoduodenectomy, commonly known as the Whipple procedure, is the surgical resection best known to resolve pancreatic cancer entails the removal of portions of the pancreas, gallbladder, duodenum, stomach and surrounding lymph nodes that contain cancer [9].

Chemotherapeutic agents used as an adjunct therapy with surgical resection have demonstrated improved mortality rates. Monotherapy with gemcitabine has long been the standard [10]. However, following two clinical trials, PRODIGE and Metastatic Pancreatic Adenocarcinoma Clinical Trial (MPACT), results showed that two combination therapies provided longer general survival rates than gemcitabine therapy only [10]. These two combination therapies have since been established as the first-line treatment for advanced pancreatic cancer, as well as adjunct to surgically resected pancreatic adenocarcinoma [10]. In the PRODIGE study, results showed the drug FOLFIRINOX (fluorouracil, leucovorin, irinotecan, and oxaliplatin) to have an overall median survival of 11.1 months compared to 6.8 months with gemcitabine used as monotherapy [10]. The MPACT study compared combination therapy of gemcitabine and nab-paclitaxel vs monotherapy of gemcitabine. The overall median survival rate was 8.5 months vs 6.7 months in combination vs monotherapy, respectively [10].

Pancreatic adenocarcinoma commonly metastasizes to nearby structures such as liver, lung, and proximal lymph node networks. Many of the metastatic sites are based on tumor staging, which is named from T1-T4. Pancreatic adenocarcinoma at T1-T2 stage is most often invaded in local lymph nodes [11]. Pancreatic adenocarcinoma with a stage T3 or greater frequently has distant metastasis and the most common sites are para-aortic and perineural lymph nodes [11].

## Conclusions

Invasion of pancreatic adenocarcinoma to the splenic flexure is a rare disease presentation which poses complex hurdles attributable to poorly understood and diverse etiologies. Diagnostic shortcomings and lack of screening methods prevent clinicians from discovering the disease early, resulting in poor prognosis and a high mortality rate. Due to the anatomic position of the pancreas and its proximity to organs in the abdominal cavity, invasion to the liver, spleen, stomach, intestines, and lymphatic system may result. Furthermore, preferred treatment consists of surgical intervention followed by monotherapy with gemcitabine regimen. 
